# Evaluation of Health Hazard and Governance Performance of Air Pollution: A Case Study of Z City

**DOI:** 10.3389/fpubh.2022.827713

**Published:** 2022-04-25

**Authors:** Ying Cui, Yunxia Shi

**Affiliations:** ^1^School of Management, Henan University of Technology, Zhengzhou, China; ^2^School of Management, Shandong Technology and Business University, Yantai, China

**Keywords:** air pollution, health hazard, economic losses, governance performance, public health

## Abstract

Based on the date of the main pollutant discharge and the change of pollutant concentration in Z city, this study performed the statistical analysis of the concentration data in excel. The data covers 3 years from 1 January 2015 to 31 December 2017. An exposure-response relationship model was used established to evaluate the health hazards caused by air pollution and the corresponding economic losses, further analyzing the relationship between air pollution, health hazards, and economic benefits. The results of the study showed that the changes in SO_2_ and NO_2_ concentrations in Z City from 2015 to 2017 have a great influence on the mortality rate of the local population, respiratory mortality, the rate of internal medicine of outpatients, and the rate of chronic bronchitis disease. The economic losses between 2015 and 2017 caused by PM10, the primary pollutant in the air, were 3.9 billion, 3.5 billion, and 2.9 billion respectively, accounting for 3.60%, 2.88%, and 2.09% of Z City's GDP, which were enormous. Finally, countermeasures of feasible treatment and in government performance were put forward.

## Introduction

Since the time of reform and the opening up in China, Z City, as known as “Greentown,” has developed prosperously with its unique geographical advantages and convenient transportation advantages. However, in recent years, the smog has shrouded the “Greentown,” and Z City has been suffering from air pollution for a long time. Air pollution not only seriously affected the physical and mental health of the residents in Z City but also caused certain economic losses, influenced the prosperous and contented life of citizens. Therefore, it is necessary to study and evaluate the health hazards and economic losses caused by the air pollution in Z City.

## Literature Review

### Health Hazard Evaluation of Air Pollution

Taking many foreign scholars' researches as examples, in Austrian schoolchildren, Horak et al. (1) discussed the evidence of adverse effects of certain air pollutants on health. They thought particulate matter and ozone exposed in the air are related to the increasing mortality rate of respiratory and cardiovascular diseases and the in-patient number.

Kim and Kim (2) linked environmental air pollution with children's poor health, and their study found that air pollution not only has an impact on preterm birth but also has a certain relationship with infant mortality. He also proposed advice on air pollution to the government to promote effective protection of children's health.

Cesaroni et al. (3) believed that the increased non-accidental mortality was associated with long-term exposure to NO_2_ and PM2.5, and long-term exposure to air pollution has a greater impact on cardiovascular, respiratory, and lung cancer mortality than other causes of death. Regarding the risk relationship between related lung cancer mortality of PM2.5 and PM10, Cui et al. (4) showed that the increased risk of getting lung cancer is related to prolonged exposure to environmental particulate matter (PM).

Through many years of research, Jiang et al. (5) pointed out that China and western developed countries have certain differences in air quality, and cannot directly use western evaluation criteria to evaluate the health hazards caused by air pollution in China.

In the long-term research, the data conforming to the status quo of China's air quality were explored gradually, which led to a theoretical foundation and significant reference for evaluating and analyzing the health hazards and economic losses caused by China's air pollution.

Liu et al. (6) studied the relationship between PM10 and PM2.5 in air and the health problems, such as acute mortality, respiratory disease incidence, and cardiovascular mortality in the population, and improved the research on health hazard evaluation of air pollution in China.

Chen et al. (7) studied and evaluated the damage caused by the air pollutant PM10 to the health of residents in 113 major cities in China and provided a reference for evaluating the health hazards of residents in other Chinese cities.

### The Research on Economic Losses Caused by the Air Pollution

Jakarta is one of the most polluted cities in the world, and its air pollution exceeded the safety limitation set by the World Health Organization. Napitupulu et al. (8) pointed out that it was estimated that the health losses caused by air pollution in Jakarta in 1999 amounted to 220 million US dollars. Therefore, the government planned to launch a plan to control vehicle emissions in 2001. Air pollution, while affecting people's physical and mental health, also increases defensive medical expenditures, directly or indirectly, and causes certain losses to people's social welfare.

Forslund et al. (9) provided a theoretical framework for measuring welfare and estimated that the negative health effects of nitrogen dioxide emissions account for 0.6% of Sweden's GDP. Research has also shown that 65% of taxes currently on nitrogen dioxide in Sweden were reasonable.

To evaluate the socio-economic impact of air pollution, Nam et al. (10) proposed a comprehensive evaluation method based on computable general equilibrium (CGE). A study of 18 Western European countries showed that the annual air pollution damage in Europe in 2000 was about 220 billion euros (about 3% of total consumption). Compared to the rest of the world, even the places where air quality was relatively high, the health damage caused by air pollution was still enormous.

Kan et al. (11) studied the health effects of air pollution on Shanghai residents and evaluated the economic losses caused by air pollution. Studies have shown that the economic losses caused by PM10 on the health of Shanghai residents in 2001 were 5.15 billion yuan (RMB), accounting for 1.03% of Shanghai's GDP.

It can be seen that the economic loss caused by air pollution in developed cities was still very serious. Hu (12) and Zhang (13) studied the impact of air pollution on human health in Qingdao and Lanzhou, respectively. The human capital method was used to estimate the economic losses caused by air pollution in the two cities, which were 353 million yuan (RMB) and 474 million yuan (RMB), respectively. From the perspective of regional space, it can be found that air pollution has caused considerable economic losses to both the eastern and western cities of China. It can be said that the span of air pollution in China is very huge, and the economic losses caused are also considerably large.

Lv and Li (14) studied the health and economic losses caused by PM10 and PM2.5 pollution in the Beijing–Tianjin–Hebei region. The results showed that the total health and economic losses caused by PM10 and PM2.5 pollution to Beijing-Tianjin-Hebei were 139.93 billion yuan (RMB) and 134.29 billion yuan (RMB), and these losses accounted for 2.26 and 2.16% of the GDP of the Beijing–Tianjin–Hebei region in 2013, respectively. It can be seen that atmospheric particulate pollution has caused great economic losses to the residents of the Beijing–Tianjin–Hebei region, of which PM10 was especially more harmful.

Generally speaking, air pollution directly or indirectly caused economic losses to China's regional development, and these losses cannot be underestimated. It is imperative to control air pollution and reduce economic losses.

### Research on Government Performance

Performance management is an important management tool for businesses and government departments. Kloot and Martin (15) pointed out that the driving force for public sectors around the world to reform is focused on the measurement of public sector organization performance, especially for local governments. Local governments are more focused on primary goals and outcomes and they lack attention to the decisive factors of secondary goals and organizational performance. Bell et al. (16) identified that accountability results can help decision-makers design the most effective air quality control policy, but in many cases, such research was hampered by enormous uncertainties. It can be seen that the accountability system has an important impact on the manager's decision-making. Although it faces certain challenges, it is still an important way of performance management. The contemporary performance system is gradually improving, but it is extraordinarily complicated.

Robinson and Moynihan (17) discussed the tense relationship between performance systems and the complexity of modern governance and identified the meaning and issues of research and practice. Low carbon governance is one of the crucial ways of air pollution control.

Wang (18) believed that on the one hand, China lacks an evaluation index system for low-carbon governance performance currently, especially for local government. On the other hand, low carbon governance is a dynamic process, but for local government's low carbon governance performance, China's dynamic evaluation is still insufficient.

In addition, the essential differences between different local governments in performance appraisal should be considered. Finally, the factors affecting the performance of local governments on low carbon governance should take into account the external environmental factors and the factors of the government organization itself. For many years, China's air pollution was dominated by smog. The long-term mechanism for seeking to manage the smog crisis is the focus of research of experts and scholars.

Meng (19) put forward the governance performance countermeasures of the local government to control air pollution by studying the work of air pollution control by Shangqiu Municipal Government of Henan Province from the aspects, such as government's strengthening joint prevention and control, vigorously developing public transportation, and controlling pollutant emission.

After reviewing some of the literature, it was found that most of the literature at home and abroad have certain similarities in the evaluation of health hazards and economic losses due to air pollution. There are various measurements to control air pollution. More attention must be attached to air pollution control.

## Research on the Current Situation of Air Quality in Z City

This study firstly introduced the development courses of Z City and then analyzed the situation of pollutant discharge and the variation trend of concentration of major pollutants, and at last analyzed the current situation of Z City's air quality and the causes of pollution.

### General Conditions of Z City

#### Geographical Environment

Geographically, Z City is located between 112°42′ and 114°14′ east longitude, 34°16′ and 34°58′ north latitude to the north of central Henan Province. It is located in the southern part of the North China Plain and downstream of the Yellow River. The terrain is high in the southwest and low in the northeast. It is situated in the transition zone between the second and third steps of China's landform.

#### Climate Environment

Z City belongs to the northern temperate continental monsoon climate with four distinctive seasons. The annual average temperature is around 15.6°C; the hottest month is August and the monthly average temperature is 27.3°C; January is the coldest month, with the monthly average temperature of 0.2°C. The annual average rainfall is 640.9 mm and the frost-free period lasts for 209 days. The annual sunshine time is about 1,869.7 hours.

#### Social Environment

The total area of Z City is 7,446 square kilometers, with 6 districts, 1 county, and 5 county-level cities under the administrative jurisdiction. According to the Statistics Bureau of Z City, the resident population of Z City in 2017 was 98.81 million, with a total production value of 136.27 billion US dollars. The per Capita GDP is $13,902. Among them, the added value of the primary industry was 2.33 billion US dollars, the added value of the secondary industry was 56.67 billion US dollars, and the added value of the tertiary industry was 62.1 billion US dollars. In the whole year, the industrial added value of industrial enterprises above the designated size decreased by 13.2%, and the comprehensive energy consumption was 17.853 million tons of standard coal.

**Figure 1:**
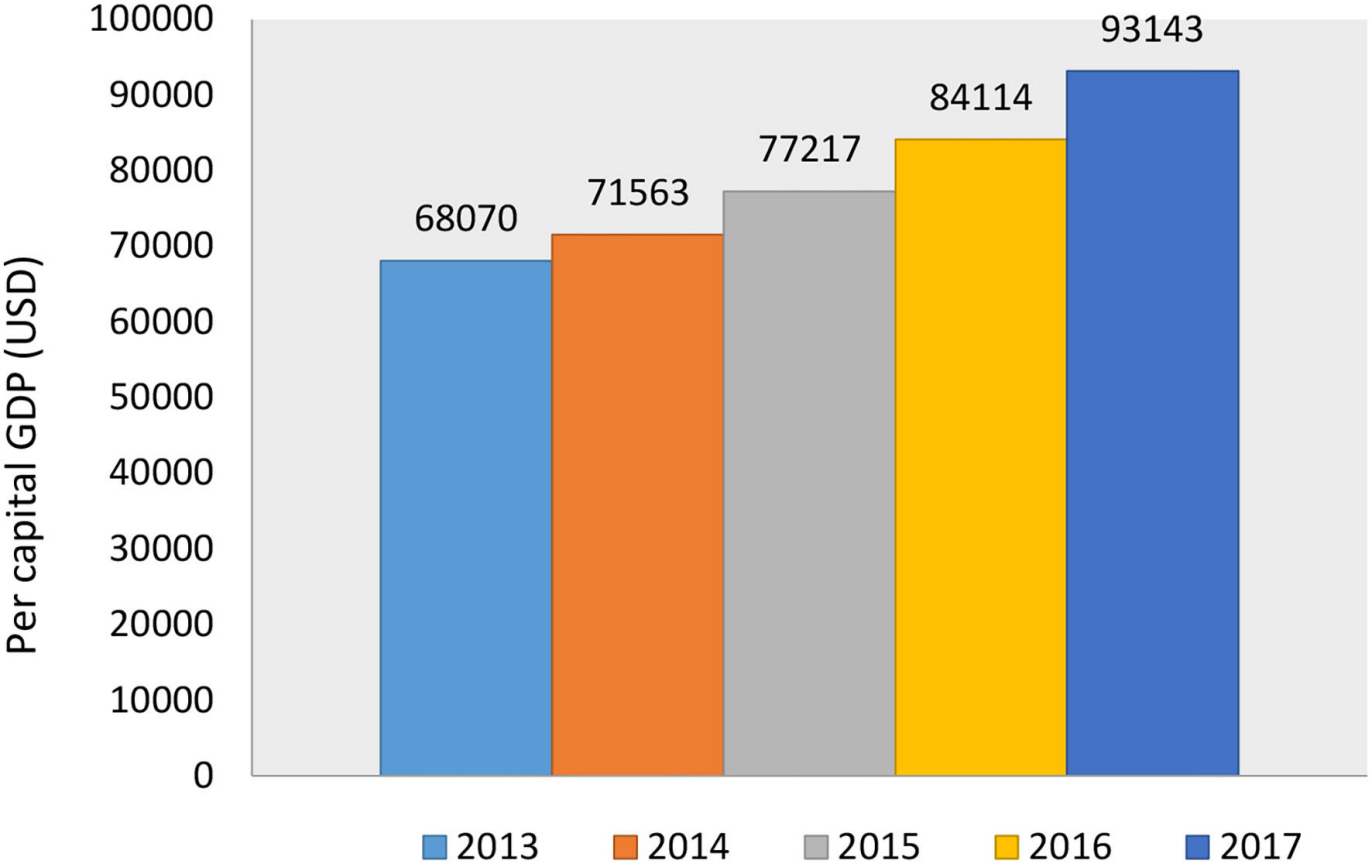
Z City's per capital GDP trends from 2013 to 2017.

To understand better the economic development of Z City in the past 5 years, this study derived the relevant data through Z City's Statistical Year Book and statistics of the GDP per capita from 2013 to 2017 and analyzed the economic development trend of Z city more intuitively, which is shown in Figure 1 below.

In Figure 1, the per capita GDP of Z City from 2013 to 2017 was $10,160, $10,681, $11,525, $12,554, and $13,902, respectively. We can see that in these 5 years, the GDP per capita of Z City increased yearly, and the amount of growth from 2013 to 2017 was 521 US dollars, 844 US dollars, 1,029 US dollars, and 1,348 US dollars, respectively. Among them, the annual growth rate in 2014 was relatively small, which was increased by only 5.10% over 2013 and the largest growth rate was in 2017, which had risen by 10.73% more than the previous year. Obviously, the overall economic development of Z City in the mentioned 5 years was excellent.

### Analysis of the Current Situation of Z City's Air Quality

#### Overview of Major Pollutant Emissions

This study firstly analyzed the annual emissions of sulfur dioxide, nitrogen oxides, and smoke (powder) dust in Z City based on environmental statistics' annual reports and identified the source of air pollutants. See Table 1 for details.

**Table 1:** Major pollutant emissions of Z City from 2011 to 2016 (Unit: ton).

**Years** **pollutants**	**2011**	**2012**	**2013**	**2014**	**2015**	**2016**
Sulfur dioxide	116,870.4	119,453	118,100	104,621.6	94,882.61	22,942.71
Nitrogen oxides	210,232.5	216,123	197,400	178,315.2	152,484.90	33,813.90
Smoke (Powder)	53,011.64	39,007.9	48,756.85	62,591.46	67,189.03	21,096.99
dust						

**Figure 2:**
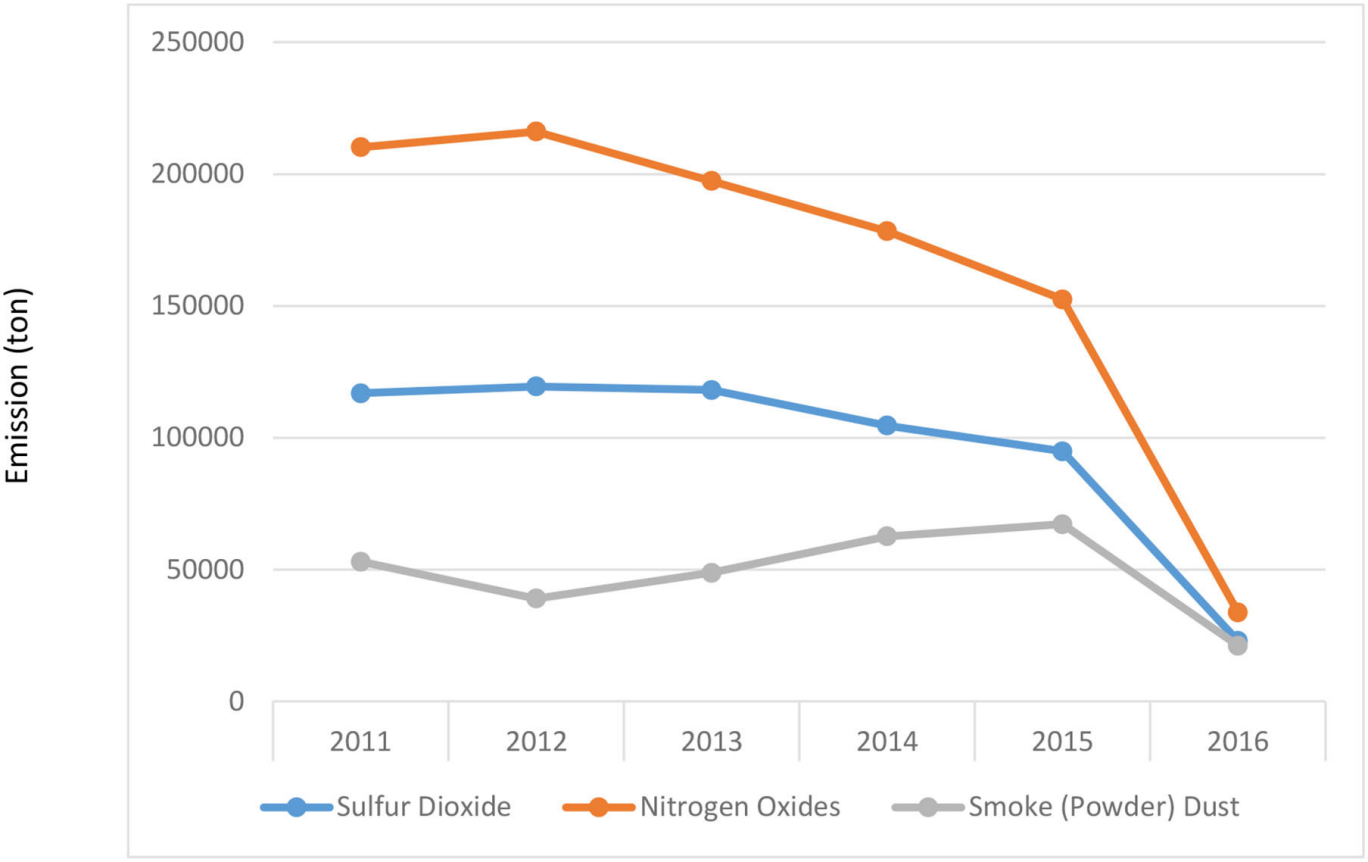
Interannual variations of major pollutant emissions in Z City from 2011 to 2016.

To analyze the annual trend of emissions of major pollutants in Z City more intuitively, a line chart of pollutant emissions was drawn in accordance with Table 1, as shown in Figure 2.

It can be seen from Figure 2 that the main pollutants of Z City were nitrogen oxides from 2011 to 2016. The emissions of nitrogen oxides in Z City increased in 2011 and 2012 and showed a downward trend for four consecutive years after 2013. The secondary pollutant was SO_2_ and the emissions were relatively stable from 2013 to 2015. In 2012, its emissions began to decline year by year. Although the emission of smoke (powder) dust was the lowest in the past years, it showed a growth trend from 2013 to 2015. There was a dramatic downward trend till 2016. The emissions of nitrogen oxides, sulfur dioxide, and smoke (powder) dust all dropped dramatically in 2016 which were very close, and according to the data, they all had dropped below 50,000 tons. This is because in 2016, Z City had strictly controlled the discharge of major pollutants and achieved good results, which was conducive to improving the status of Z City's air quality.

#### Analysis of the Annual Atmospheric Change Trend of Pollutant Concentration

From Table 2, the concentration of PM10 in Z City's atmosphere from 2013 to 2017 was 171, 158, 164, 143, and 118 μg/m^3^ respectively, which were far above the standard of the air quality of China and did not conform to the requirements for the secondary concentration limitation of 70 μg/m^3^, which was specified in *Ambient air quality standards (GB 3095-2012)*.

**Table 2:** Concentration of atmospheric particulate pollutants of Z City from 2013 to 2017 (Unit: μg/m3).

**Years** **pollutants**	**2013**	**2014**	**2015**	**2016**	**2017**
PM10	171	158	164	143	118
PM2.5	108	88	93	78	66
SO_2_	58	41	32	28	20
NO_2_	51	49	54	53	52

During the 5 years, the concentrations of PM2.5 were 108, 88, 93, 78, and 66 μg/m^3^ respectively, which also failed to meet the requirements of *Ambient air quality standards (GB 3095-2012)*, according to which the secondary concentration limitation of PM2.5 should be 35 μg/m^3^. While, the concentration of SO_2_ in these 5 years were 58, 41, 32, 28, and 20 μg/m^3^ respectively, which reached the second level specified in *Ambient air quality standards (GB 3095-2012)*, according to which the secondary level concentration limitation of SO_2_ should be 60 μg/m^3^. In 2017, the concentration of SO_2_ was almost up to the first level concentration limitation.

The concentration of NO_2_ in Z City's atmosphere from 2013 to 2017 were 51, 49, 54, 53, and 52 μg/m^3^, respectively, which were above the secondary concentration limitation of NO_2_ of 40 μg/m^3^ specified in *Ambient air quality standards (GB 3095-2012)* in China. In summary, the main pollutants in Z City's atmosphere were PM10, PM2.5, NO_2_, and SO_2_. These pollutants posed a potential threat to the physical and mental health of the citizens of Z City. It can be seen that the current situation of Z City's air quality was still not optimistic. Controlling air pollution is a long-term process, which needs the public to pay more attention to and never slacken off.

**Figure 3:**
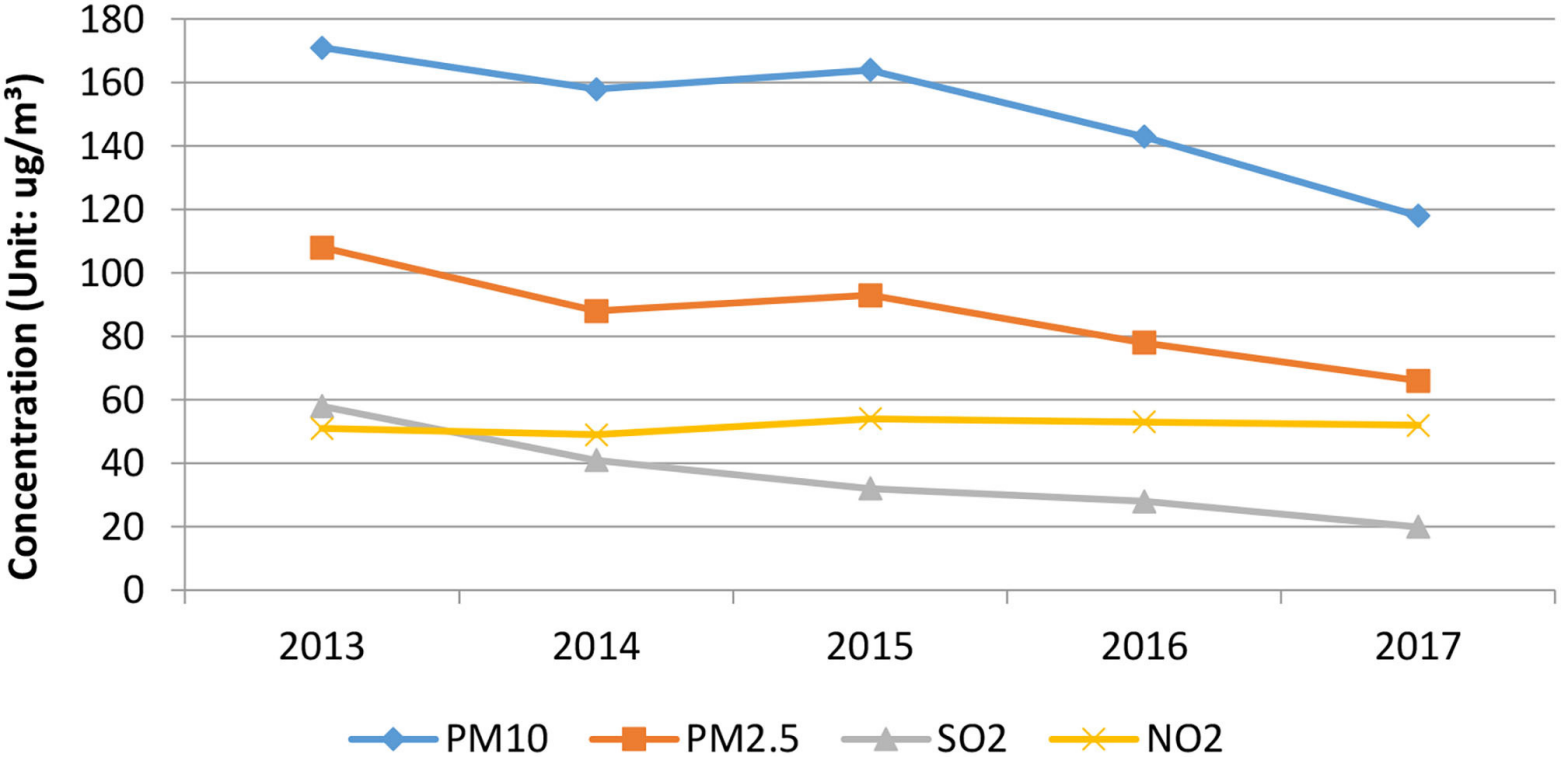
Interannual variations trends of the concentration of inhalable particulate matter in Z City from 2103 to 2017.

To be more intuitive in the analysis of the Interannual variations of major pollutants in Z City, the annual trend of each pollutant was plotted according to Table 2, as shown in Figure 3 below.

From Figure 3, we can find out that the trends of the concentrations of PM10 and PM2.5 in Z City were approximately the same from the year 2013 to 2017. Among the inhalable particulate matter in Z City in 2013, the concentrations of PM10 and PM2.5 was the highest in 5 years; in 2014, the concentration was decreased; in 2015, it rebounded and showed an upward trend; since 2016, the concentration of inhalable particulate matter decreased for two consecutive years. The concentration change of NO_2_ in Z City from 2013 to 2017 was relatively stable, but it has shown a trend of slow increase in the past 3 years, indicating that the emission of NO_2_ in Z City has increased in the past 3 years because fewer control measures were taken. In the past 5 years, the concentration of SO_2_ in Z City has shown a downward trend, especially in 2014, when it declined the most and the concentration dropped by about 29% compared with the concentration in 2013. It can be seen that although the air quality of Z City has shown an improving trend in recent years, the air pollution was still very serious, and the effectiveness of air pollution control was still not stable. The road ahead for air pollution control in Z City is still long and tough.

#### Analysis of the Causes of Air Pollution in Z City

##### Climatic Factor

Z City has a marked continental monsoonal climate and it is in the mid-latitude region. The four seasons are distinct and the temperature is moderate. It is hot and rainy in summer and cold and dry in winter. In winter, the winds are less, lacks rain and snow, is prone to inversion weather, and the air fluidity is poor, which is not conducive for the diffusion of pollutants, resulting in their accumulation.

##### Social Factors

On the one hand, Z City's energy structure is dominated by coal, accounting for about 73% of energy consumption; on the other hand, Z city has a large number of cars and large vehicle exhaust emissions; in addition, the density of population and construction in Z City is relatively high, and all these factors make the air liquidity weaken and aggravate air pollution. Furthermore, other pollutants, such as factory exhaust gas and construction site dust, have aggravated air pollution.

#### Analysis of the Physical Health Status of Z City Residents

**Figure 4:**
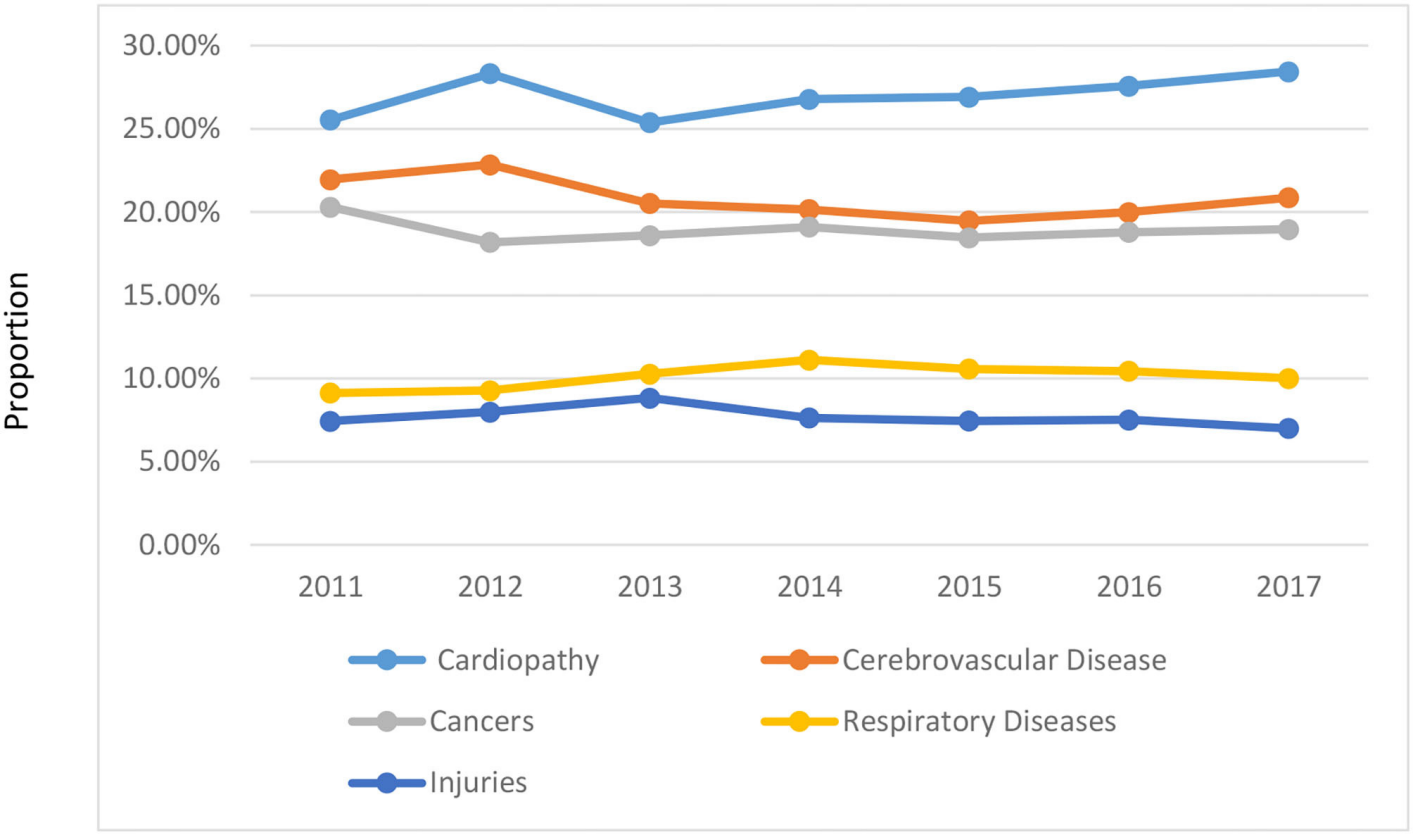
The Trends of death cause of Z City population.

To evaluate the relationship between air pollution in Z City and its impact on the residents' health, it is necessary to understand the health status of Z City residents. This study searched the archived death-related data of the residents through the network and analyzed the causes of death from 2011 to 2017. The causes and dynamic trends of residents in Z City are shown in Table 3 and Figure 4.

**Table 3:** Statistics on the causes of death of Z City residents.

**Years** **causes**	**2011**	**2012**	**2013**	**2014**	**2015**	**2016**	**2017**
Cardiopathy	25.54%	28.32%	25.38%	26.78%	26.91%	27.57%	28.44%
Cerebrovascular disease	21.95%	22.85%	20.52%	20.16%	19.47%	19.99%	20.86%
Cancers	20.30%	18.19%	18.59%	19.11%	18.46%	18.79%	18.96%
Respiratory diseases	9.13%	9.28%	10.27%	11.11%	10.57%	10.44%	10.01%
Injuries	7.44%	7.99%	8.83%	7.64%	7.45%	7.52%	7.01%

It can be seen from Table 3 and Figure 4 that from 2011 to 2017 cardiopathy has been the main cause of death in Z city. It has been increasing year by year since 2013. Cerebrovascular disease is the second leading cause of death and cancers are the third. The trends of these three causes of death have been the same basically since 2013. Respiratory diseases are the fourth leading cause of death, and their trends are relatively stable. The proportion of people who died from injuries in Z City was relatively small. In conclusion, the cause of death in Z City is mainly due to cardiopathy, cerebrovascular disease, and cancer, which is not related to the lifestyle, but related to their living environment.

## Empirical Study on the Relationship Among Air Pollution, Health of Residents, and Economic Losses of Z City

In this study, the exposure–response relationship model was used to evaluate the harm caused by air pollution to the physical health of residents in Z City in 2015, 2016, and 2017. Based on the outputs, we combined it with the economic value of the unit health effect in known research and the corresponding impact on residents' health and economic losses caused by air pollution was calculated further.

### Research Model Settings

#### Health Hazard Assessment Methods

In this study, the exposure–response relationship model derived from epidemiological studies at home and abroad was used to evaluate the health threats caused by air pollution in Z city. The exposure–response relationship model is associated with the change in air quality and adverse health outcome, which is the key to quantitatively assess the health hazards caused by air pollution. Since the epidemiological study of air pollution is based on the proportional hazard model of Poisson regression, assuming the population health effect value at a certain particle concentration is:


(1) $$\begin{array}{r} \text{E}=\text{exp}\left[\text{β}\times \left(\text{C}-{\text{C}}_{0}\right)\right]\times E_{0} \end{array}$$

(2) $$\begin{array}{r} \text{E}=\text{P}\times \text{e} \end{array}$$

In the equations

β—The coefficient relationship of Exposure-reaction between particulate contaminants and a certain adverse health outcome, which refers to each change in particulate matter.

1 ug/m3, The increased proportion of the adverse health outcome.

C—Actual concentration of particulate matter, unit: ug/m3.

C_0_—Threshold concentration value without health effect, unit: ug/m3.

E–Population health effects under polluted conditions unit: case(s).

E_0_—Population health effects under clean conditions unit: case(s).

P—The number of the exposed population, that is, the number of permanent residents in the study year unit: person(s).

e—Baseline incidence: the average incidence of individual the adverse health outcome in the population, units: 1/100,000.

ΔE—The health effect attributed to atmospheric particulate contamination, i.e., the difference between E and E0.

#### Estimation Method of Economic Loss

At present, the estimation of economic losses of atmospheric particulate pollution mainly includes the Willingness to Pay Method, the Human Capital Method, and the Disease Cost Method. Multiplying the unit value of the adverse health outcome by the corresponding health effect is the economic loss of the population in the adverse health outcome. The calculation model used in this study is as follows:


(3) $$\begin{array}{r} \text{E}{\text{C}}_{\text{al}}=P_{\text{ed}}^{*}{\left(E-E_{0}\right)}^{*}HC_{\text{mu}} \end{array}$$

In Equation (3)

EC_al_—Economic losses arising from residents' health hazards resulted from atmospheric particulate pollutants, unit: Yuan.

P_ed_—The total number of exposed population, unit: Person (s).

HC_mu_—Unit economic value of each adverse health outcome, unit: Yuan.

### Analysis of Health Hazard of Air Pollution

#### Data Source

##### Exposed Population

In this study, the population in Z City belongs to the exposed population. However, due to the large population flow in Z City, the number of permanent residents in Z City from 2015 to 2017 was taken as the exposed population for the assessment of the health hazard caused by atmospheric particulate pollution in the calculation. The specific data comes from the Z City Statistical Yearbook of Z City. From 2015 to 2017, the resident population of Z City was 9,569,000; 9,724,000; and 9,881,000, respectively.

##### Exposure–Response Relationship

The exposure–response relationship correlates the change in particulate matter pollution with the change in adverse health outcomes, which is the key to quantitatively evaluate the health hazard of particulate matter pollution. The selection of exposure–response relationship coefficient β has to take into account the sensitivity of race and ethnic groups, the atmospheric particulate matter pollution, and the difference in the degree of air pollution at home and abroad, and these should be analyzed by using epidemiological data in China. Only when there is no relevant information in China, similar research data in other countries would be substituted.

##### Selection of Pollution Factors

Since the concentration of CO and O^3^ in Z City from 2015 to 2017 was lower than the first-level concentration limitation of each pollutant specified in *Ambient air quality standards (GB 3095-2012)*, its impact on human health is not big. Therefore, this study selected PM2.5, PM10, SO_2_, and NO_2_ as the main pollution factors when evaluating the health hazards caused by atmospheric pollutants in the urban residents of Z City.

##### Threshold Concentration Value

This study uniformly uses the first-level concentration limitation specified in *Ambient air quality standards (GB 3095-2012)* as the evaluation reference value for the health damage and value assessment of air pollutants in Z City. The first-level concentration limitation of PM10, PM2.5, SO_2_, and NO_2_ are 40, 15, 20, and 40 ug/m^3^, respectively. The actual pollutant concentration can be obtained through relevant statistics and weather reports.

##### Adverse Health Outcome

It relatively lacks data on the baseline incidence rate of the Z City's population and unit economic value of adverse health outcomes after comprehensively considering the current epidemiological research status in China combined with the actual situation of the Z City. The study evaluated the following six adverse health outcomes: total mortality, respiratory mortality, in-patient number of cardiovascular disease, the number of patients with chronic bronchitis, number of patients with asthma, and the number of internal medicine of outpatients.

##### Baseline Incidence Rate and Correlation Coefficient

Since the health baseline data of residents of Z City was difficult to obtain, some data in this study uniformly adopted the adverse health outcome average rate of Chinese urban residents.

#### Evaluation Steps

The correlation coefficient and the baseline incidence of each adverse health outcome of Z City is shown in Table 4 (20–25).

**Table 4:** Exposure-response relationship coefficient and baseline incidence of relevant adverse health outcomes.

**The adverse health outcomes**	**Exposure-response relationship coefficient (Average and 95%CI)**	**Baseline Incidence (e)**
Total mortality	0.00430 (0.00260, 0.00610)	0.00522
Respiratory mortality	0.01430 (0.00850, 0.02010)	0.00042
The in-patient number for cardiovascular disease	0.00070 (0.00031, 0.00109)	0.01428
The number of patients with chronic bronchitis	0.00450 (0.00127, 0.00773)	0.00148
The number of asthma patients	0.02100 (0.01450, 0.03000)	0.00627
The number of internal medicine of outpatients.	0.00090 (0.00012, 0.00168)	0.5156

According to Equation (2), the baseline incidence rate “e” of Z City and the annual total population “P” (the total population data involved in this study have been given in the exposed population data) can be used to calculate the baseline number “E” of the next adverse health outcomes at the annual contaminant concentration of Z City from 2015 to 2017. The result is shown in Table 5.

**Table 5:** Baseline number of each adverse health outcome from 2015 to 2017 [unit: case (s)].

**Adverse health outcomes**	**2015**	**2016**	**2017**
Total mortality	49,950	50,759	51,579
respiratory mortality	4,019	4,084	4,150
The in-patient number for cardiovascular disease	136,645	138,859	141,101
The number of patients with chronic bronchitis	14,162	14,392	14,624
The number of asthma patients	59,998	60,969	61,954
The number of internal medicine of outpatients.	4,933,776	5,013,694	5,094,644

It can be seen from Table 5, among the number of baseline occurrences of the six diseases caused by atmospheric particulate matter pollution in Z City from 2015 to 2017, the number of internal medicine of outpatients was the largest, which was related to the baseline incidence. The second is the in-patient number for cardiovascular disease, the third is the number of patients with asthma, the total mortality rate and chronic bronchitis cases are fourth and fifth, respectively, and respiratory mortality is at the sixth place.

#### Analysis of Results

According to Equation (1), the population health effect value was calculated under the threshold concentration of urban residents of Z City from 2015 to 2017. The calculated baseline occurrence number “E” and the population health effect value “E${}_{0}^{{}^{\prime \prime }}$ under the threshold concentration was used to obtain the attribution occurrences number “ΔE” of adverse health outcomes of Z City, where the difference between the baseline occurrence number and health effect value at the threshold concentration is equal to ΔE, and the calculation result is shown in Table 6 as follows:

**Table 6:** City health loss caused by pollutants in Z City (cases).

**Main pollutants**	**Year**	**Total mortality**	**Respiratory mortality**	**The in-patient number for cardiovascular disease**	**The number of patients with chronic bronchitis**	**The number of asthma patients**	**The number of internal medicine of outpatients**
PM2.5	2015	14,233	2,702	7,261	4,192	48,336	334,474
	2016	12,046	2,425	5,991	3,553	44,731	276,367
	2017	10,157	2,149	4,948	2,999	40,724	228,559
PM10	2015	20,643	3,337	11,361	6,056	55,559	520,997
	2016	18,163	3,148	9,659	5,338	53,959	443,878
	2017	14,697	2,790	7,498	4,329	49,912	345,379
SO_2_	2015	2,512	634	1,143	744	13,365	52,998
	2016	2,076	14	14,991	169	2,983	4,932,639
	2017	0	0	0	0	0	0
NO_2_	2015	2,918	729	1,333	865	15,283	61,776
	2016	2,760	693	1,258	818	14,566	58,318
	2017	2,594	654	1,180	769	13,801	54,726

As can be seen from Table 6, the total number of deaths in Z City caused by PM2.5 was 14,233; 1,2046; and 10,157, respectively. The number of respiratory deaths was 2,702; 2,745; and 2,149; respectively. The in-patient number for cardiovascular disease was 7,261; 5,991; and 4,948, respectively. The number of patients with chronic bronchitis was 4,192; 3,553; 2,999, respectively. The number of asthma patients was 48,336; 44,731; and 40,724, respectively. The number of internal medicine of outpatients was 334,474; 276,367; and 228,559, respectively. Among them, the number of internal medicine outpatients is the largest, which means, the incidence influenced by PM2.5 is the highest in the general population. Generally speaking, the incidence of adverse health outcomes affected by PM2.5 showed a trend of decreasing year by year, which was mainly related to the change in PM2.5 concentration in Z City. It can be seen that there is a certain correlation between air pollution in Z City and the health hazard to the residents.

From 2015 to 2017, the number of internal medicine of outpatients in Z City was the largest among the six diseases caused by PM10, followed by the number of patients with asthma. The third was the total number of deaths, the in-patient number for cardiovascular disease and chronic bronchitis cases was fourth and fifth, and respiratory mortality was at the sixth place. Taking the year 2017 as an example, the number of internal medicine of outpatients was 345,379, the number of patients with asthma was 49,912, and the total number of deaths was 14,697. The three adverse health outcomes were in the top three. Comparing them with the annual trend of the number of patients from various adverse health outcomes from 2015 to 2017, the prevalence rate of various diseases in Z City has generally declined year by year, that is to say, the air quality of Z city has improved in the past 3 years.

From 2015 to 2017, the adverse health outcomes of Z City have varying levels of sensitivity to SO_2_. The total number of deaths caused by SO_2_ in Z city was 2,512; 2,076; and 0, respectively. The number of respiratory deaths was 634, 14, and 0, respectively. The in-patient number for cardiovascular disease was 1,143; 14,991; and 0, respectively. The case of chronic bronchitis was 744, 169, and 0, respectively. The case of asthma was 13,365; 2,983; and 0, respectively. The number of internal medicine of outpatients was 52,998; 4,932,639; and 0, respectively. In summary, with the decline of SO_2_ concentration in Z City from 2015 to 2017, the number of each adverse health outcome affected by SO_2_ also showed a trend of decreasing year by year. The various health losses caused by SO_2_ in 2017 are all 0 because the SO_2_ concentration in Z City was equal to the threshold concentration value, which was just up to the first level of air quality standard in China.

From 2015 to 2017, the total number of deaths caused by pollutants in Z City was 2,918; 2,760; and 2,594, respectively. The number of respiratory deaths was 729, 693, and 654, respectively. The in-patient number in hospitals for cardiovascular disease was 1,333; 1,258; and 1,180, respectively. The number of chronic bronchitis was 865, 818, and 769, respectively. The cases of asthma were 15,283; 14,566; and 13,801, respectively. The number of internal medicine outpatients was 61,776; 58,318; and 54,726, respectively. In summary, the number of adverse health outcomes affected by NO_2_ also decreased with the decrease of NO_2_ concentration in Z City.

Taking the internal medicine outpatients caused by various pollutants in 2017 as an example, 228,559 cases were caused by PM2.5, 345,379 cases were caused by PM10, 0 cases were caused by SO_2_, and 54,726 cases were caused by NO_2_. It can be seen that PM10 among the four pollutants is the most harmful to the health of Z City citizens, followed by PM2.5; NO_2_ is ranked the third, and SO_2_ is the fourth.

For example, the concentrations of PM10, which is the most harmful to human health, in Z City were 164, 143, and 118 ug/m^3^, respectively from 2015 to 2017. The total population was 9,569,000; 9,724,000; and 9,881,000, respectively. The death toll was 44,000, 53,000, and 55,000, respectively. The total death toll of the population attributed to PM10 was 20,643; 18,163; and 14,697, respectively. That is to say, among the 44,000 deaths in 2015, 20,643 deaths were related to the pollutant PM10. Similarly, 18,163 deaths in the 2016 death toll were associated with PM10, and in 2017, 14,697 deaths were associated with it. The number of internal medicine outpatients caused by PM10 from 2015 to 2017 was 520,997; 443,878; and 345,379, respectively, which also showed a downward trend. Obviously, the number of adverse health outcomes affected by PM10 showed a decreasing trend, which was basically consistent with the downward trend of PM10 concentration.

### Research on Economic Loss Caused by Air Pollution

#### Data Source

On the basis of the existing research results for calculating the economic loss of air pollution in China, we use the same method adopted by the World Bank combined with the relevant statistical data of the General Survey of Health Services in China, and this study calculated the unit value of each adverse health outcome caused by air pollution in Z City and assessed economic losses.

#### Evaluation Steps

The method adopted in this study, the willingness to pay method, monetized the pollution hazard of major pollutants in air and was combined with the disease cost method to supplement the adverse health outcomes lacking partial willingness of paying price.

Table 7 summarized the adverse health outcomes associated with atmospheric particulate matter pollution in Z City, the evaluation methods used, and the unit economic value of the disease.

**Table 7:** Economic value and evaluation method of each adverse health outcome in Z City (Unit: ten thousand dollars).

**The adverse health outcome**	**Unit economic value /ten thousand Yuan**	**The evaluation methods adopted**
Total mortality rate	14.92	Willing-to-pay evaluation method
Respiratory mortality rate	13.28	Willing-to-pay method
The in-patient number for cardiovascular disease	0.16	Cost of illness evaluation method
The number of patients with chronic bronchitis	5.97	Willing-to-pay evaluation method
The number of asthma patients	0.001	Willing-to-pay method
The number of internal medicine of outpatients	0.004	Willing-to-pay evaluation method

As we can see from Table 7, urban residents are more willing to pay to prevent dying of the population and respiratory deaths, respectively, at $149,200 and $132,800, followed by chronic bronchitis, which is $59,700, while, they spent the lowest on internal medicine, only $40.

#### The Analysis of Results

Combining the data in Tables 6, 7, the study evaluated the health hazard of urban residents caused by air pollution in Z City and used Equation (3) to calculate the health economic loss values of various diseases caused by selected pollution factors in Z City. As shown in Table 8.

**Table 8:** Economic losses caused by various pollutants in Z City, from 2015 to 2017 (ten thousand) Unit: US dollar.

**Major pollutants**	**Year**	**Total mortality rate**	**Respiratory mortality rate**	**The in-patient number for cardiovascular disease**	**The number of patients with chronic bronchitis**	**The number of asthma patients**	**The number of internal medicine of outpatients**	**Its proportion in GDP**
PM2.5	2015	212,436	35,887	1,127	25,028	30	1,498	2.53%
	2016	179,784	32,214	930	21,210	27	1,237	1.94%
	2017	151,595	28,542	768	17,904	25	1,023	1.47%
PM10	2015	308,107	44,322	1,763	36,158	34	2,333	3.60%
	2016	271,091	280,151	1,499	31,869	33	1,987	2.88%
	2017	219,362	41,814	1,164	25,844	31	1,546	2.09%
SO_2_	2015	37,493	8,418	177	4,445	8	237	0.47%
	2016	30,986	182	2,327	1,011	2	22,086	0.47%
	2017	0	0	0	0	0	0	0
NO_2_	2015	43,556	9,686	207	5,162	9	277	0.54%
	2016	41,188	9,203	195	4,882	9	261	0.46%
	2017	38,716	8,693	183	4,590	8	245	0.38%

It can be seen from Table 8 that the changing trend of the economic losses caused by various pollutants and the health effects were basically the same. According to the statistics of the Statistics Bureau, the GDP of Z City from 2015 to 2017 was 109.18 billion US dollars, 121.1 billion US dollars, and 136.27 billion US dollars, respectively. The economic losses caused by various pollutants in Z City were as follows: the economic losses caused by PM2.5 accounted for 2.53%, 1.94%, and 1.47% of the GDP of the year, respectively; the economic losses were 3.9 billion US dollars, 3.5 billion US dollars, and 2.9 billion US dollars, respectively, accounting for 3.60, 2.88, and 2.09% of the GDP of the year, respectively; the health losses caused by SO_2_ accounted for 0.47, 0.47, and 0% of the GDP of the year, respectively; the economic losses caused by NO_2_ accounted for 0.54, 0.46, 0.38% of the GDP in the year. Overall, the economic losses caused by PM10 to Z City were greater than other pollutants, while the economic losses caused by SO_2_ are relatively small, especially in 2017, and there were no losses caused by SO_2_

In general, with the improvement of air quality in Z City, the corresponding economic losses also decreased accordingly. Therefore, air pollution has a certain negative impact on the economic development of Z City, and the economic losses on urban transportation and construction caused by air pollution cannot be underestimated at the same time. It is imperative to control air pollution and alleviate the economic losses of Z City.

## Government Performance on Air Pollution Control in Z City

In recent years, because of the efforts from many parties, the air pollution control of Z City has achieved certain results. This aspect showed that in the strategic framework of China's “Twelfth Five-Year Plan,” the municipal government of Z City developed the economy while vigorously improving regional environmental quality, controlling air pollution, making overall plans for regional economic, and environmental development to improve people's livelihood. Besides, Z City Government strictly carried out the requirements of the Ten Measures for Air Pollution Prevention and Control, was committed to energy conservation and emission reduction in Z City, and actively fulfilled the performance assessment task of atmospheric environment remediation. However, the control of air pollution will take a long time. It is necessary to guard against arrogance and not be in a hurry to succeed or violate the objective laws of economic development.

Based on the relevant data of the Environmental Protection Bureau of Z City and the Statistical Yearbook, this study concluded that the air quality compliance status of Z City from 2013 to 2017 reached the standard (air quality reaches the Second level or above) and analyzed the current situation of air quality control in Z City, as shown in Table 9.

**Table 9:** Air quality status of reaching the standard in Z City from 2013 to 2017.

**Year**	**2013**	**2014**	**2015**	**2016**	**2017**
Days reaching the standard (days)	128	163	138	159	201
Rate of reaching the standard (%)	35.10	44.70	37.80	43.40	55.06

It can be seen from Table 9 that it took only 128 days to reach the standard in the year 2013 in Z City, and the reaching rate was 35.10%. In 2014, the time of air quality reaching the standard in Z City was 163 days, which was 35 days more than in 2013, and the reaching rate increased by 9.6%. In 2015, the air quality of Z City deteriorated, and the time of air quality reaching the standard was only 138 days, which was 25 days less than that of 2014. In 2016, the air quality of Z City improved compared with the previous year. In 2016, the time of air quality reaching the standard was 159 days, which was 21 days longer than that in 2015, and the reaching rate increased by 15%. In 2017, the time of air quality reaching the standard in Z City was 201 days, which was 42 days more than the previous year and the growth rate was 26%. Overall, in the 5 years, Z City's air quality standards and reaching rates have shown an increasing trend, which indicates that the government and relevant departments of Z City have paid much attention to the air pollution control process and achieved certain results. Especially in 2017, the government's efforts and results in air pollution control were even more obvious than at any other year.

According to Table 9, the line chart shows that the annual variation trend of the Z City's air quality reaching rate can be analyzed more intuitively.

**Figure 5:**
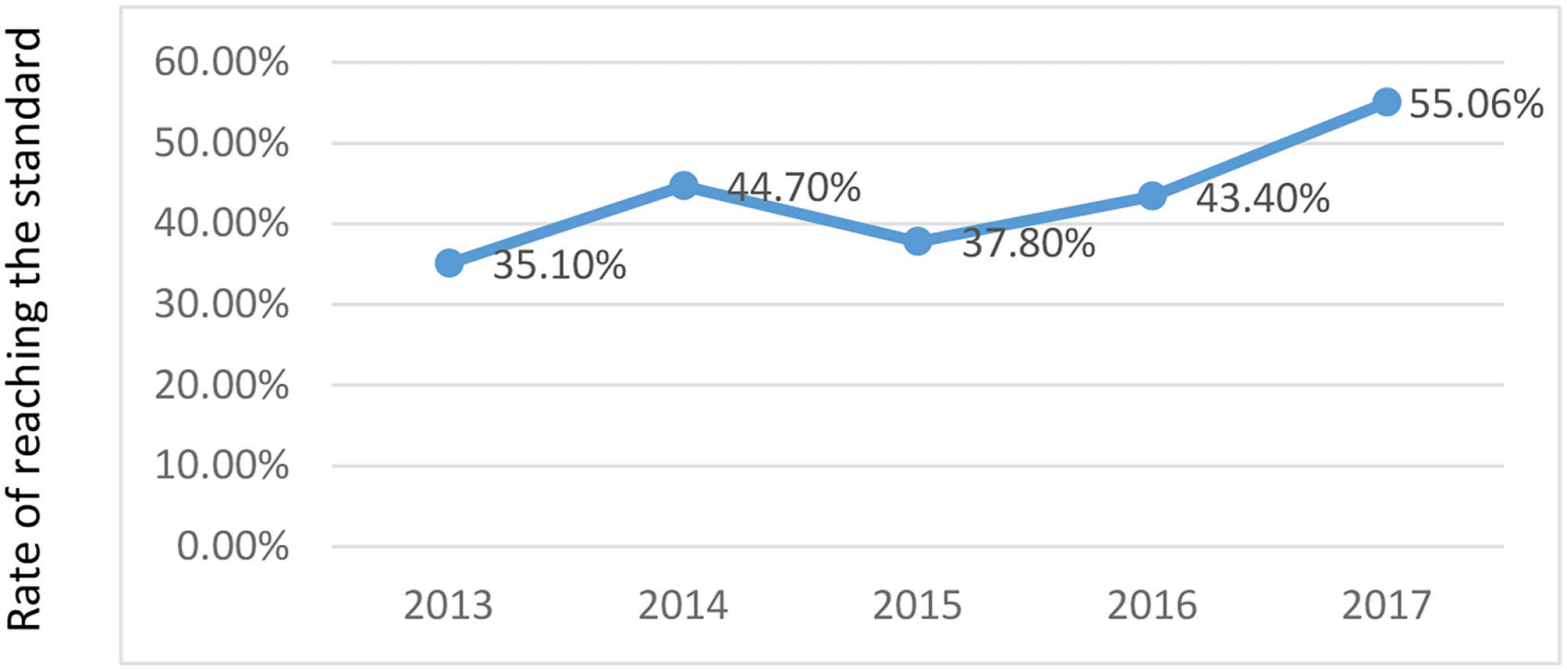
The trend of reaching standard rate of Z City's air quality.

We can see from Figure 5 that during the period from 2015 to 2017, the air quality reaching the rate of Z City has been basically an upward trend, reaching an optimal level in 2017. When the economic growth of Z City was improved during the 2 years, it also paid attention to the treatment of air quality. Therefore, the policies and regulations on air pollution control and air pollution prevention and control in the past few years have important referential significance for better prevention and control of air pollution in the future.

## Conclusion

This study comprehensively analyzed the emission of air pollutants, the variation trend of pollutant concentration, the causes of pollution in Z City, and the health status of residents in Z City, evaluated the health hazards and economic losses caused by air pollution in Z City, and helped the residents to have a deeper understanding of the harm of air pollution, and improve the awareness of self-protection. In the past few years, the air quality of Z City is worrying, but the government of Z City, the enterprises, and the people of Z City are very concerned about air pollution. With the joint efforts of all sectors of society, the emission of air pollutants in Z city has gradually decreased, the concentration of air pollutants has decreased, and the air quality compliance rate has increased year by year, showing a trend of improvement. Combined with the health damage assessment caused by air pollution in Z City, the economic damage caused by air pollution in Z city was evaluated. The research shows that the causes of air pollution in Z city are multifaceted, including geographical and meteorological factors as well as social and economic development factors. Air pollution not only brings inconvenience to people's life, production, and work, but also seriously threatens the health of urban residents, causing certain economic losses and greatly reducing the happiness of residents in Z City. Studying the current situation of air pollution control in Z City and putting forward a feasible control suggestion based on the research results contributes to improving the urban environment of Z City and promoting its economic development.

In recent years, the Z City government has adopted a series of strict control measures to strengthen the control of air pollution, which has achieved certain results. The air quality is improving day by day with a stable and positive trend, but the current results do not reach the second-level standard of the national environmental air quality standard. There is still a long way to go to fight air pollution, and the environmental movement is still in a tough position. Joint and constant efforts are highly required.

## Data Availability Statement

The original contributions presented in the study are included in the article/supplementary material, further inquiries can be directed to the corresponding author.

## Author Contributions

YC wrote the draft of the manuscript. YS contributed to manuscript revision. All authors contributed to data curation and visualization. All authors approved the submitted version.

## Funding

This study was supported by the National Social Science Foundation of China (21BGL052), Henan Philosophy and Social Science Planning Project (2019BJJ027), and Henan Science and Technology Think Tank Project (HNKJZK-2022-42B).

## Conflict of Interest

The authors declare that the research was conducted in the absence of any commercial or financial relationships that could be construed as a potential conflict of interest.

## Publisher's Note

All claims expressed in this article are solely those of the authors and do not necessarily represent those of their affiliated organizations, or those of the publisher, the editors and the reviewers. Any product that may be evaluated in this article, or claim that may be made by its manufacturer, is not guaranteed or endorsed by the publisher.
